# Effect of Thyroid-Stimulating Hormone Suppression on Muscle Function After Total Thyroidectomy in Patients With Thyroid Cancer

**DOI:** 10.3389/fendo.2021.769074

**Published:** 2021-11-10

**Authors:** Jun Choul Lee, Byong-Sop Song, Young Mi Kang, Yu-Ri Kim, Yea Eun Kang, Ju Hee Lee, Minho Shong, Hyon-Seung Yi

**Affiliations:** ^1^ Research Center for Endocrine and Metabolic Diseases, Chungnam National University School of Medicine, Daejeon, South Korea; ^2^ Department of Core Laboratory of Translational Research, Biomedical Convergence Research Center, Chungnam National University Hospital, Daejeon, South Korea

**Keywords:** thyroid-stimulating hormone, sarcopenia, thyroidectomy, thyroid cancer, muscle function and physical activity

## Abstract

**Context:**

Thyroid-stimulating hormone (TSH) suppression is recommended to reduce tumor recurrence following surgery for differentiated thyroid cancer (DTC). However, prolonged subclinical hyperthyroidism caused by levothyroxine treatment has deleterious effects on various organs.

**Objective:**

To evaluate the relationships of TSH concentration with muscle mass, muscle strength, and physical performance related to sarcopenia in patients with DTC undergoing TSH suppression following surgery.

**Methods:**

We studied 134 patients of >60 years who were undergoing TSH suppression therapy following surgery for DTC. We evaluated muscle mass and muscle function-related parameters and diagnosed sarcopenia using the threshold for Asian people.

**Results:**

The participants were 68.3 ± 7.2 years old and 36/134 (26.9%) were diagnosed with sarcopenia. They were allocated to high-TSH and low-TSH groups using a threshold concentration of 0.40 μU/mL, and grip strength was significantly lower in the low-TSH group. The data were further analyzed according to age and sex, and in the low-TSH group, male participants and those of <70 years were found to have significantly lower grip strength.

**Conclusions:**

Low-TSH concentrations is associated with low grip strength, and this is most pronounced in individuals of <70 years of age. Therefore, muscle function should be considered an adverse effect of TSH suppression in patients with DTC who undergo TSH suppression therapy, especially in men of <70 years.

## Introduction

The standard treatment for differentiated thyroid cancer (DTC) consists of thyroid surgery, with or without postoperative radioiodine therapy and thyroid-stimulating hormone (TSH) suppression therapy, according to the estimated risk of recurrence (1–3). For those treated using thyroidectomy and for some who undergo thyroid lobectomy alone, TSH suppression therapy, usually conducted alongside levothyroxine administration, is necessary to restore euthyroidism and is used to inhibit cancer recurrence, which suggests that DTCs are TSH-dependent tumors (4–6). Because aggressive TSH suppression therapy is of little or no benefit to most patients with DTC, levothyroxine is not routinely administered following unilateral lobectomy. Furthermore, clinical guidelines recommend that the oncologic benefits of TSH suppression are weighed against the cardiovascular and musculoskeletal risks (7).

Sarcopenia is defined as an age-related, involuntary, progressive loss of muscle mass and strength, and it affects approximately 50% of adults aged ≥80 years (8). Sarcopenia is frequently accompanied by long-standing physiologic consequences of metabolic disorders and malignancies in patients, and together these have various adverse sequelae, including physical disability, poor quality of life, and death (9, 10). Patients with advanced thyroid cancer who are treated using molecularly targeted therapies often experience a significant loss of skeletal muscle mass, irrespective of the rate of disease progression (11), but no previous studies have evaluated the relationships of thyroid hormone or TSH concentration with sarcopenia in patients with indolent DTC.

Hyperthyroidism and hypothyroidism affect multiple body systems, including skeletal muscle, because this is a principal target tissue of thyroid hormones. Although thyroid hormone excess in overt hyperthyroidism induces osteoporosis and myopathy, and impairs physical performance, subclinical hyperthyroidism, defined as a TSH concentration below the normal range, alongside normal serum thyroxine concentration, is also associated with bone loss and a higher risk of fracture (12, 13). Although most studies have shown the effects of overt or subclinical hyperthyroidism on the heart or bone tissue, there is no research on the effects of subclinical hyperthyroidism on physical and functional muscle profiles and sarcopenia incidence in patients with DTC. Furthermore, it remains to be elucidated how excessive T4 supplementation-induced subclinical hyperthyroidism affects muscle mass and function after total thyroidectomy in differentiated thyroid cancer patients. In addition, recent studies have shown that the TSH receptor is expressed in skeletal muscle cells (14, 15). TSH also directly modulates muscle metabolism, independently of thyroid hormone, as has been demonstrated with respect to bone remodeling (16). Therefore, we hypothesized that, even though the role of TSH receptor signaling in skeletal muscle is poorly understood, TSH suppression may be associated with higher risks of the loss and/or dysfunction of muscle in patients with DTC who are administering levothyroxine. In the present study, we aimed to evaluate the relationship of TSH concentration with muscle mass, muscle strength, and physical performance in older patients with DTC.

## Materials and Methods

### Study Participants

We performed a cross-sectional study of a sample of Korean patients of >60 years of age who were undergoing TSH suppression therapy following surgery for DTC between April 2019 and April 2020. These participants were attending the Outpatient department of the hospital for the management of DTC and TSH suppression therapy. Anthropometric measurements were made after an overnight fast. Height (in centimeters) and body mass (in kilograms) were measured, and body mass index (BMI) was calculated as body mass divided by the square of height (kg/m^2^). Individuals with liver cirrhosis, renal failure, stroke sequelae, myocardial infarction, or angina were excluded because these may affect muscle metabolism. After the exclusion of ineligible individuals, 134 eligible participants were enrolled.

### Ethical Considerations

The Institutional Review Board (2019-06-063) of Chungnam National University Hospital approved the research protocol. Written informed consent was obtained from all the participants. The interviews and examinations were performed in accordance with the principles of the Declaration of Helsinki. The authors certify their compliance with the ethical guidelines for authorship and publishing in the journal.

### Assessment of Sarcopenia

Information regarding the demographic characteristics and medical and surgical histories of the participants was collected through detailed interviews and reviews of medical records by experienced nurses. Body composition, including muscle mass (whole-body lean mass minus bone mineral content), was evaluated using a bioelectrical impedance analyzer (InBody S10; InBody, Seoul, Korea) at frequencies of 1, 5, 50, 250, 500, and 1,000 kHz (17). Appendicular skeletal muscle mass (ASM) was calculated as the sum of the muscle mass of all four limbs. Skeletal muscle mass index (SMI) was calculated as ASM/height^2^ (kg/m^2^) (18). Hand-grip strength on the dominant side was measured using a Jamar hydraulic hand dynamometer (Patterson Medical, Warrenville, IL, USA) (19). Participants were instructed to sit comfortably, bend their elbow to 90°, and grip the dynamometer as firmly as possible. The maximum value was recorded after all the tests were conducted twice at 1 min intervals or more. We also measured gait speed over a 4 m distance and the time taken to complete five chair-stands (20). The participants also underwent a short physical performance battery (SPPB), which consisted of repeated chair-stands, and assessments of balance when standing and gait speed (21). In the standing balance test, which comprised side‐by‐side, semi-tandem, and tandem stances, the participants were instructed to stand for up to 10 sec. Higher SPPB scores (range 0 to 12 points) are indicative of superior function of the lower extremities.

Sarcopenia was diagnosed using the 2019 Consensus Guidelines of the Asian Working Group for Sarcopenia (22). Briefly, older patients with low muscle mass (SMI <7.0 kg/m^2^ for men and <5.7 kg/m^2^ for women) and low muscle strength (hand-grip strength <28 kg for men and <18 kg for women), and/or poor physical performance (gait speed <1.0 m/s, five-time chair-stand test ≥12 s, or SPPB score ≤9 points) were diagnosed as having sarcopenia.

### Thyroid Function and Biochemical Measurements

Blood samples were collected from an antecubital vein and centrifuged at 400 g for 5 min at 4°C, and then the supernatants were carefully collected. Samples showing hemolysis or clotting were discarded. The serum samples were stored at −80°C until analyzed. Serum TSH and free T4 (FT4) concentrations were measured using electrochemiluminescence immunoassays (Roche Diagnostics, Mannheim, Germany) 24 h after sampling. Serum TSH was measured using an E-TSH kit (Roche Diagnostics; reference range: 0.35–5.50 mIU/L) and serum FT4 was measured using an E-Free T4 kit (Roche Diagnostics; reference range: 0.89–1.76 ng/mL).

### Statistical Analysis

Clinical data are presented as means ± standard deviations (SDs) or as numbers and percentages unless otherwise specified. The chi-square and Fischer’s exact tests were used to analyze categorical data. The normality of continuous variables was assessed using the Shapiro-Wilk test, and homogeneity of variance was assessed using Levene’s test. If the normality and homogeneity of variance assumptions were satisfied, then independent *t*-tests were used to compare the means of two groups and ANOVA was used to compare the means of three groups. If the normality assumption but not the homogeneity of variance assumption was satisfied, unpaired *t*-tests and Kruskal-Wallis tests were substituted. If neither assumption was satisfied, Mann-Whitney *U*-tests and Kruskal-Wallis tests were used for continuous clinical and biochemical data, as appropriate. A two-tailed *p* < 0.05 was considered to represent statistical significance.

Statistical analyses were performed using R software version 4.0.4 (R Project for Statistical Computing, Vienna, Austria).

## Results

### Baseline Characteristics of the Study Participants

**Supplementary Table 1** lists the baseline characteristics of the 134 study participants, of whom 109 (81.3%) were women, and the mean ± SD age was 68.33 ± 7.19 years. Their mean serum free T4 concentration was 1.42 ± 0.25 ng/mL and their mean TSH concentration was 0.84 ± 0.98 μIU/mL during thyroid function testing. Their mean BMI was 24.32 ± 3.24 kg/m^2^, their mean skeletal muscle mass was 21.54 ± 4.53 kg, and their mean SMI was 8.68 ± 1.15 kg/m^2^. According to the 2019 Consensus Guidelines of the Asian Working Group for Sarcopenia, 36 of the 134 (26.9%) participants were diagnosed with sarcopenia. Their mean grip strength, an index of muscle strength, was 21.54 ± 5.40 kg. With respect to indices of physical performance, their mean gait speed was 4.34 ± 1.38 m/s, their mean five-time chair-stand test result was 8.57 ± 3.52 s, and their mean SPPB score was 11.04 ± 1.75 points.

### Effects of Age on Muscle Function in Older Patients Undergoing Total Thyroidectomy

We allocated the participants to two groups on the basis of age (**Table 1**): an over-70s group (n=55) and an under-70s group (n=79). There were 66 (83.5%) and 43 (78.2%) women in each group, respectively (*P* = 0.433). The mean FT4 concentrations were 1.38 ± 0.26 ng/mL in the over-70s group and 1.45 ± 0.23 ng/mL in the under-70s group (*P* = 0.059). The mean TSH concentrations were 0.74 ± 0.96 μIU/mL in the over-70s group and 0.91 ± 0.99 μIU/mL in the under-70s group (*P* = 0.530). Twenty-three participants (41.8%) were diagnosed with sarcopenia in the over-70s group and 13 (16.5%) in the under-70s group (*P* = 0.001). However, there were no differences in skeletal muscle mass or SMI between the two groups (*P* = 0.095 and 0.212, respectively). Moreover, there were no differences in grip strength or the five-time chair-stand test result between the two groups (*P* = 0.325 and 0.115, respectively), but the gait speed in the over-70s group (4.78 ± 1.33 s) was higher than that in the under-70s group (4.03 ± 1.34 s) (*P* = 0.002). There was also a lower SPPB score in the over-70s group (10.40 ± 2.20 points) than in the under-70s group (11.49 ± 1.18 points) (*P* = 0.001).

**Table 1:** Clinical characteristics of the study sample, categorized according to age (N = 134).

Parameter	60-70 years (N = 79)	>70 years (N = 55)	*P-*value
Sex			0.577
Male	13 (16.5)	12 (21.8)	
Female	66 (83.5)	43 (78.2)	
Sarcopenia			**0.002**
Absent	66 (83.5)	32 (58.2)	
Present	13 (16.5)	23 (41.8)	
Body mass index (kg/m^2^)	24.22 ± 3.39	24.47 ± 3.02	0.502
Skeletal muscle mass (kg)	21.92 ± 4.66	20.98 ± 4.31	0.095
Skeletal muscle index (kg/m^2^)	8.75 ± 1.22	8.59 ± 1.04	0.212
Free T4 (ng/mL)	1.45 ± 0.23	1.38 ± 0.26	0.059
T3 (ng/mL)	1.39 ± 0.19	1.36 ± 0.19	0.664
TSH (µIU/mL)	0.91 ± 0.99	0.74 ± 0.96	0.530
Grip strength (kg)	21.99 ± 5.48	20.91 ± 5.25	0.325
Chair-stand test result (s)	8.17 ± 3.47	9.21 ± 3.52	0.115
B-score	3.99 ± 0.11	3.91 ± 0.35	0.113
Chair score	3.76 ± 0.69	3.62 ± 0.74	0.269
Gait speed (s)	4.03 ± 1.34	4.78 ± 1.33	**0.002**
Gait speed score	3.84 ± 0.52	3.46 ± 0.79	**0.003**
SPPB score	11.49 ± 1.18	10.40 ± 2.20	**0.001**

Data are mean ± SD or number (%). The chi-square or t-tests were used to compare the groups, as appropriate. Significant differences between the age groups are highlighted in bold.

TSH, thyroid stimulating hormone; BUN, blood urea nitrogen; eGFR, estimated glomerular filtration rate; HDL, high-density lipoprotein; SPPB, short physical performance battery.

### Relationship Between Serum TSH Concentration and Muscle Function in Older Patients Undergoing Total Thyroidectomy

Next, we allocated the participants to a high-TSH group (0.40–4.0 μU/mL; n = 69) and a low-TSH group (<0.40 μU/mL; n = 65) (**Table 2**). There was no significant differences in the sex ratio or the prevalence of sarcopenia between the high-TSH (73.9%) and low-TSH groups (72.3%) (*P* = 0.988). In addition, there were no differences between the groups with respect to the results of the five-time chair-stand test, gait speed, or SPPB score (*P* = 0.295, 0.297, and 0.612, respectively). However, hand-grip strength was significantly lower in the low-TSH group (20.30 ± 3.96 kg) than in the high-TSH group (22.72 ± 6.27 kg) (*P* = 0.007). Logistic regression analysis was performed to confirm the relationship between hand-grip strength and TSH, and the Akaike information criterion (AIC) was used to assess a model’s maximum likelihood estimation. The AIC of the logistic model was 172.9. There was an odds ratio of 1.1563 for grip strength for the high-TSH group, meaning that the probability of being in the high-TSH group increased 1.1563 times for each increase of 1 in grip strength (**Table 3**). However, there was no relationships of free T4 or T3 concentrations with muscle function or physical performance in older patients who had undergone total thyroidectomy (**Supplementary Tables 2**, **3**).

**Table 2:** Clinical characteristics of the study sample, categorized according to serum TSH concentration (N = 134).

Parameter	<0.40 µU/ml (N = 65)	≥0.40 µU/ml (N = 69)	*P-*value
Sex			0.244
Male	9 (13.8)	16 (23.2)	
Female	56 (86.2)	53 (76.8)	
Sarcopenia			0.988
Absent	47 (72.3)	51 (73.9)	
Present	18 (27.7)	18 (26.1)	
Age (years)	68.42 ± 7.45	68.25 ± 6.99	0.641
Body mass index (kg/m^2^)	24.24 ± 3.07	24.40 ± 3.41	0.780
Skeletal muscle mass (kg)	21.09 ± 3.81	21.96 ± 5.10	0.428
Skeletal muscle index (kg/m^2^)	8.59 ± 0.92	8.77 ± 1.33	0.423
Free T4 (ng/mL)	1.52 ± 0.27	1.33 ± 0.19	**0.000**
T3 (ng/mL)	1.42 ± 0.21	1.34 ± 0.16	0.126
Grip strength	20.30 ± 3.96	22.72 ± 6.27	**0.007**
Chair-stand test result (s)	8.91 ± 3.44	8.24 ± 3.58	0.295
B-score	3.95 ± 0.28	3.96 ± 0.21	0.949
Chair score	3.67 ± 0.73	3.75 ± 0.70	0.538
Gait speed (s)	4.21 ± 1.07	4.46 ± 1.62	0.297
Gait speed score	3.68 ± 0.64	3.69 ± 0.70	0.903
SPPB score	11.02 ± 1.74	11.07 ± 1.78	0.612

Data are mean ± SD or number (%). The chi-square or t-tests were used to compare the groups, as appropriate. Significant differences between the TSH groups are highlighted in bold.

TSH, thyroid stimulating hormone; BUN, blood urea nitrogen; eGFR, estimated glomerular filtration rate; HDL, high-density lipoprotein; SPPB, short physical performance battery.

**Table 3:** Results of the logistic regression analysis of potential predictors of serum TSH concentration.

No interaction (AIC = 172.9)			
Parameter	OR	2.50%	97.50%
(Intercept)	1.4385	0.0066	328.5889
Sarcopenia	1.4095	0.4244	4.7348
Grip strength (kg)	1.1563	1.0444	1.2945
Age (years)	0.4897	0.2105	1.1024
Free T4 (ng/mL)	0.1646	0.0657	0.3818
Skeletal muscle (kg)	1.1047	0.8679	1.4087
SPPB score	0.9153	0.6653	1.2496
Skeletal muscle index (kg/m^2^)	0.6526	0.2744	1.5235

SPPB, short physical performance battery.

### Subgroup Analyses of the Data on the Bases of Age and Sex

**Figure 1:**
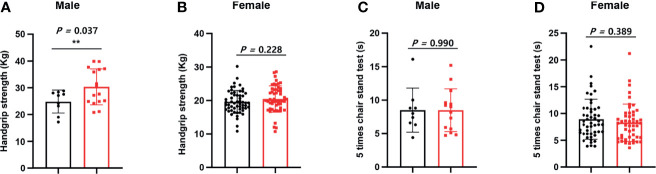
Relationships of grip strength and chair-stand test result with serum TSH concentration and sex. **(A)** We allocated the participants to two groups on the basis of sex. Grip strength in men was significantly lower in the low-TSH group than in the high-TSH group. **(B)** However, grip strength in women was similar in the high-TSH and low-TSH groups. **(C)** The results of the chair-stand test were analyzed in the same way. The results of the test were similar in men in the high-TSH and low-TSH groups. **(D)** The results of the test were also similar in women in the high-TSH and low-TSH groups. ** in the figure indicates p-value < 0.01.

**Figure 2:**
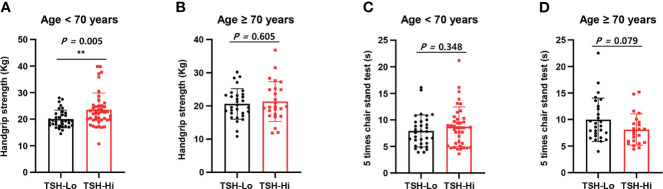
Relationships of grip strength and chair-stand test results with serum TSH concentration and age. **(A)** Participants were allocated to two groups on the basis of age: <70 years and >70 years. Grip strength in the under-70s was significantly lower in the low-TSH group (TSH-Lo, <0.40 μU/mL). **(B)** Grip strength in the over-70s was similar in the high- (TSH-Hi, 0.40–4.0 μU/mL) and low-TSH groups. **(C)** The chair-stand test results were similarly analyzed. Among the under-70s, there was no difference between the low- and high-TSH groups. **(D)** Among the over-70s, there was also no difference between the low- and high-TSH groups. ** in the figure indicates p-value < 0.01.

**Figure 3:**
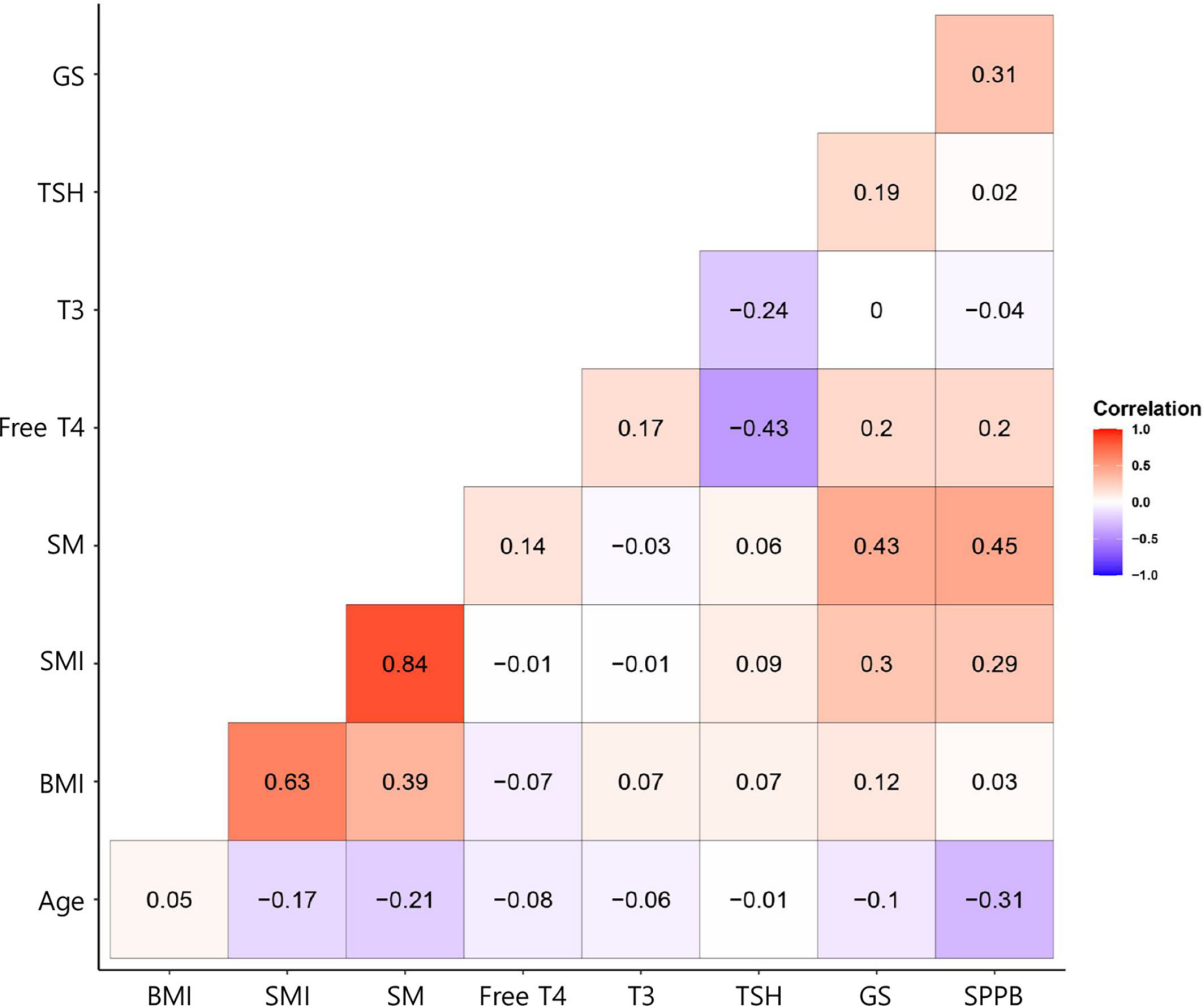
Correlation coefficients for the relationships between explanatory variables and the response variable for the participants with thyroid cancer. In the corrgram, the depth of shading at the correlation matrices indicates the magnitude of the correlation. Positive and negative correlations are represented in red and blue, respectively.

As shown in **Figure 1**, we calculated correlations to characterize the relationships among all the clinical variables. Grip strength tended to be lower in participants of <70 years and in men with a low-TSH concentration. The correlation coefficient for the relationship between TSH concentration and grip strength was 0.19, implying a weak positive correlation between the two variables (**Figure 1**). We next determined whether grip strength is related to age or sex in participants with a low-TSH concentration. First, we allocated the participants to two groups on the basis of being <70 or >70 years old, and then further allocated them to high- and low-TSH groups. In the over-70s group, there was no difference in hand-grip strength between the high- and low-TSH groups, but in the under-70s group, grip strength was significantly lower in participants with low-TSH concentration than in those with high-TSH (**Figure 2**). However, there were no relationships between five-time chair-stand test performance and serum TSH concentration in either the over-70s or under-70s groups. Next, the participants were categorized according to sex and the muscle function of each sex was compared between low- and high-TSH groups. There was no difference in grip strength according to TSH concentration in women, but it was significantly lower in men with a low-TSH concentration. As for the results of the analysis according to age, there was no relationship between five-time chair-stand performance or TSH concentration in either men or women (**Figure 3**).

## Discussion

In the present study, we have compared the prevalence of sarcopenia, muscle strength, and physical performance in patients with DTC who had high or low serum TSH concentrations. We found that those with low-TSH concentrations who were 60–70 years old had lower grip strength than those who were > 70 years old.

The degree of TSH suppression in patients with DTC should be optimized to reduce tumor recurrence while minimizing the risk of toxicity associated with subclinical hyperthyroidism. TSH suppression significantly increases the postoperative risks of atrial fibrillation and osteoporosis in patients with DTC (23), and subclinical hyperthyroidism is associated with higher cardiovascular morbidity and mortality in older patients with DTC (24, 25). Furthermore, postmenopausal women with DTC and subclinical hyperthyroidism are at higher risk of osteoporosis, whereas the risks for men and premenopausal women are not affected by this (26). Therefore, we believe that measurement of bone mineral density should be recommended for postmenopausal women with DTC when TSH is being suppressed. The American Thyroid Association recommends that serum TSH should be suppressed to low concentrations (0.1–0.5 mU/L) for 5–10 years in high-risk groups only (3). However, although the major academic societies have made recommendations regarding TSH suppression-related adverse effects on heart and bone, on the basis of published evidence, the optimal maintenance TSH concentration for the preservation of muscle strength and physical performance in patients with DTC has not been determined. Therefore, our finding that TSH suppression may have adverse effects on skeletal muscle function in elderly patients with DTC is of great relevance. Moreover, we suggest that subclinical hyperthyroidism may be implicated as a modifier of muscle function in individuals with total thyroidectomy of <70 years of age.

Sarcopenia, which is associated with both low absolute muscle mass and poor muscle function, is a problem in elderly patients and increases in prevalence with age. Moreover, the prevalence of DTC also increases with age. Therefore, the effects of postoperative thyroid function on muscle function and physical performance in our aging societies are important. We have studied older adults with DTC (age 68.33 ± 7.19 years) and found a prevalence of sarcopenia of 26.9%, according to the diagnostic criteria of the Asian Working Group (22). This prevalence of sarcopenia in older patients of DTC is comparable with that of community-dwelling individuals of >65 years when SMI (ASM/height^2^) is used as an index of sarcopenia, with thresholds of 7.09 kg/m^2^ for men and 5.27 kg/m^2^ for women (27). To evaluate the relationship of age with TSH suppression-related muscle function, we further allocated the participants to 60–70 years old and > 70 years old groups. As expected, the prevalence of sarcopenia in the latter group (41.8%) was higher than that in the former group (16.5%). Intriguingly, we found that TSH suppression was associated with low hand-grip strength in the 60–70-year-old participants but not in the older participants. This suggests that the frailty of the older patients may conceal or prevent TSH suppression-related muscle deterioration. Therefore, we further considered the roles of potential risk factors for frailty: age, sex, ethnicity, nutritional status, polypharmacy, educational level, cognitive function, marital status, living status, drinking and smoking status, regular exercise, and self-reported health. We found that more marked TSH suppression was associated with lower hand-grip strength, but only in men. Serum TSH concentrations are considered the most reliable indicator of thyroid function abnormalities, and TSH analysis stands as the primary means of studying thyroid function (28). In contrast, free T4 assays often fail to be reliable due to variable TBG and albumin levels (29–31). This may explain why only low TSH concentrations, not free T4 or free T3, were associated with low grip strength in this study. However, these findings need to be confirmed in a large cohort study using methods that take into account the effects of confounding factors.

Grip strength is a measure of the maximum static force that the hand can exert on a dynamometer and is a reliable index of overall muscle strength (32, 33). Low grip strength is associated with comorbidities such as hypertension, diabetes, cardiovascular disease, stroke, and chronic obstructive pulmonary disease, as well as high all-cause mortality (34–39). Therefore, grip strength has been suggested to represent a “biomarker of aging” across the lifespan (40, 41). Importantly, low grip strength is a clinical marker of poor mobility and a better predictor of clinical outcomes than low muscle mass (42, 43). Moreover, muscle strength is not solely dependent on muscle mass, and the relationship between muscle strength and mass is not always linear (44, 45). Therefore, careful consideration of the importance of muscle strength *per se* and muscle mass is necessary in research studies and in the clinic to better understand the effects of particular factors on muscle health. In the present study, we have shown that a low-TSH concentration is associated with low grip strength in older patients with DTC. Although we believe that TSH may regulate the biogenesis and molecular function of skeletal muscle cells (14), further experimental studies are required to define the effects of TSH on muscle physiology and pathology in the context of aging.

The present study had several limitations. First, because most of the participants were elderly, there are likely to be various factors that would have affected their muscle metabolism and function; therefore, it was not possible to assess the pure relationship between TSH and muscle function. Given the potential limitations of the observational studies and the marginally statistically significant association, it is difficult to determine between TSH and muscle parameters using the conclusion of this study. Moreover, our study population was exclusively South Korean, and we cannot be certain that our results apply to other populations. Furthermore, we excluded the participants with liver cirrhosis, renal failure, stroke sequelae, myocardial infarction, or angina in the current study, but other confounders, such as respiratory disease, autoimmune disease, uncontrolled diabetes, low calcium intake and vitamin D level, sex hormone level, and statin use, should also be excluded to enhance statistical significance. In addition, we studied a relatively small number of patients in a single institution. Therefore, further studies should be conducted using a larger sample size and over a wider area.

In conclusion, we have shown that a low TSH concentration is associated with low grip strength, especially in individuals of <70 years of age and in men. Therefore, clinicians should be aware of the adverse effects of TSH suppression on muscle function in patients with DTC who undergo TSH suppression therapy, especially if they are male and under 70 years old.

## Supplemental Methods

### Statistical analysis

To identify factors affecting TSH concentration, logistic regression analysis was performed using age, sex, SMI, free T4, T3, sarcopenia, grip strength, SPPB score, gait speed, and skeletal muscle mass as explanatory variables. Because low-TSH and high-TSH represent a binary response variable, we used the Logit-model. Finally, Akaike’s information criterion and multicollinearity among the explanatory variables were considered in the selection of the final model.

## Data Availability Statement

The raw data supporting the conclusions of this article will be made available by the authors, without undue reservation.

## Ethics Statement

The studies involving human participants were reviewed and approved by Chungnam National University Hospital. The patients/participants provided their written informed consent to participate in this study.

## Author Contributions

Conception or design: H-SY and MS. Acquisition, analysis, or interpretation of data: JCL, B-SS., YMK, Y-RK, YEK, and JHL. Drafting the work or revising: JCL and H-SY. Final approval of the manuscript: H-SY. All authors contributed to the article and approved the submitted version.

## Funding

This work was supported by the Basic Science Research Program through the National Research Foundation of Korea (NRF), funded by the Ministry of Science, ICT, and Future Planning, Korea (NRF-2021R1A5A8029876), and by the Chungnam National University Hospital Research Fund, 2021. H-SY was supported by a grant from the Korea Health Technology R&D Project, through the Korea Health Industry Development Institute, funded by the Ministry of Health & Welfare, Republic of Korea (grant number: HR20C0025), and by the Korean Endocrine Society, through a Hyangseol Young Investigator Award (2020).

## Conflict of Interest

The authors declare that the research was conducted in the absence of any commercial or financial relationships that could be construed as a potential conflict of interest.

## Publisher’s Note

All claims expressed in this article are solely those of the authors and do not necessarily represent those of their affiliated organizations, or those of the publisher, the editors and the reviewers. Any product that may be evaluated in this article, or claim that may be made by its manufacturer, is not guaranteed or endorsed by the publisher.

## Supplementary Material

Supplementary Table 1 Clinical characteristics of the study sample (N = 134). Data are mean ± SD or number (%). TSH, thyroid stimulating hormone; BUN, blood urea nitrogen; eGFR, estimated glomerular filtration rate; HDL, high-density lipoprotein; SPPB, short physical performance battery.

Supplementary Table 2 Clinical characteristics of the study sample, categorized according to serum FT4 concentration (N = 134). Data are mean ± SD or number (%). The chi-square or *t*-tests were used to compare the groups, as appropriate. Significant differences between the FT4 groups are highlighted in bold. TSH, thyroid stimulating hormone; SPPB, short physical performance battery.

Supplementary Table 3 Clinical characteristics of the study sample, categorized according to serum T3 concentration (N = 134). Data are mean ± SD or number (%). The chi-square or *t*-tests were used to compare the groups, as appropriate. TSH, thyroid stimulating hormone; SPPB, short physical performance battery.
