# Supplementary material for: Effect of Thyroid-Stimulating Hormone Suppression on Muscle Function After Total Thyroidectomy in Patients With Thyroid Cancer
Source: Front Endocrinol (Lausanne). 2021 Nov 10;12:769074. doi: 10.3389/fendo.2021.769074 (PMC8631429; doi:10.3389/fendo.2021.769074)
Supplement: Supplementary Table 1 — Clinical characteristics of the study sample (N = 134). Data are mean ± SD or number (%). TSH, thyroid stimulating hormone; BUN, blood urea nitrogen; eGFR, estimated glomerular filtration rate; HDL, high-density lipoprotein; SPPB, short physical performance battery. [file DataSheet_1.docx]

**Supplemental Data**

**Supplemental Methods**

***Statistical analysis***

To identify factors affecting TSH concentration, logistic regression analysis was performed using age, sex, SMI, free T4, T3, sarcopenia, grip strength, SPPB score, gait speed, and skeletal muscle mass as explanatory variables. Because low-TSH and high-TSH represent a binary response variable, we used the Logit-model. Finally, Akaike’s information criterion and multicollinearity among the explanatory variables were considered in the selection of the final model.

**Supplemental Table 1. Clinical characteristics of the study sample (N = 134)**

| Characteristic | Mean±SD or number (percentage) |
| --- | --- |
| Sex  Male  Female | 25 (18.7)  109 (81.3) |
| Age | 68.33±7.19 |
| Body mass index (kg/m^2^) | 24.32±3.24 |
| [Skeletal muscle mass](https://en.dict.naver.com/#/entry/enko/2bf2da66a9ca4364a05bbc76e0eac6be) (kg) | 21.54±4.53 |
| Skeletal muscle index (kg/m^2^) | 8.68±1.15 |
| Sarcopenia  Absent  Present | 98 (73.1)  36 (26.9) |
| Free T4 (ng/mL) | 1.42±0.25 |
| T3 (ng/mL) | 1.38±0.19 |
| TSH (µIU/mL) | 0.84±0.98 |
| Aspartate transaminse (IU/L) | 25.54±7.36 |
| Alanine transaminase (IU/L) | 21.14±10.71 |
| Alkaline phosphatase (IU/L) | 67.20±23.90 |
| Total bilirubin (mg/dL) | 0.75±0.26 |
| Total protein (g/dL) | 7.07±0.38 |
| Albumin (g/dL) | 4.17±0.24 |
| Triglyceride (mg/dL) | 141.02±71.04 |
| Total cholesterol (mg/dL) | 178.48±33.36 |
| BUN (mg/dL) | 16.58±5.31 |
| Creatinine (mg/dL) | 0.74±0.22 |
| eGFR (mL/min/1.73m^2^) | 88.22±20.62 |
| Calcium (mg/dL) | 11.45±14.44 |
| Phosphorus (mg/dL) | 3.72±0.77 |
| Uric acid (mg/dL) | 4.37±1.27 |
| Glucose (mg/dL) | 112.21±21.48 |
| Sodium (mmol/L) | 140.81±2.43 |
| Potassium (mmol/L) | 4.39±0.38 |
| Chloride (mmol/L) | 103.23±2.45 |
| HDL-cholesterol (mg/dL) | 53.04±13.88 |
| Grip strength (kg) | 21.54±5.40 |
| Chair-stand test result (s) | 8.57±3.52 |
| B-score | 3.96±0.24 |
| Chair score | 3.71±0.71 |
| Gait speed(s) | 4.34±1.38 |
| Gait speed score | 3.68±0.67 |
| SPPB score | 11.04±1.75 |

Data are mean±SD or number (%). Abbreviations: TSH, thyroid stimulating hormone; BUN, blood urea nitrogen; eGFR, estimated glomerular filtration rate; HDL, high-density lipoprotein; SPPB, short physical performance battery.

**Supplemental Table 2. Clinical characteristics of the study sample, categorized according to serum FT4 concentration (N = 134**)

| Parameter | Low FT4 | High FT4 | *p-*value |
| --- | --- | --- | --- |
|  | (N=85) | (N=49) |  |
| Sarcopenia |  |  | 0.501 |
| Absent | 60 (70.59%) | 38 (77.55%) |  |
| Present | 25 (29.41%) | 11 (22.45%) |  |
| Sex |  |  | 0.122 |
| Male | 12 (14.12%) | 13 (26.53%) |  |
| Female | 73 (85.88%) | 36 (73.47%) |  |
| Age |  |  | 0.341 |
| <70 years | 47 (55.29%) | 32 (65.31%) |  |
| ≥70 years | 38 (44.71%) | 17 (34.69%) |  |
| T3 |  |  | 0.653 |
| <1.53 (ng/mL) | 70 (82.35%) | 38 (77.55%) |  |
| ≥1.53 (ng/mL) | 15 (17.65%) | 11 (22.45%) |  |
| TSH |  |  | **0.000** |
| <0.40 (µIU/mL) | 31 (36.47%) | 34 (69.39%) |  |
| ≥0.40 (µIU/mL) | 54 (63.53%) | 15 (30.61%) |  |
|  |  |  |  |
| Age | 68.69 ± 7.19 | 67.69 ± 7.21 | 0.637 |
| Body mass index (kg/m^2^) | 24.52 ± 2.95 | 23.99 ± 3.69 | 0.390 |
| Skeletal muscle mass (kg) | 21.16 ± 4.39 | 22.18 ± 4.72 | 0.213 |
| Skeletal muscle index (kg/m^2^) | 8.67 ± 1.15 | 8.71 ± 1.15 | 0.908 |
| T3 (ng/mL) | 1.36 ± 0.19 | 1.40 ± 0.19 | 0.136 |
| TSH (µIU/mL) | 1.09 ± 1.03 | 0.42 ± 0.72 | **0.000** |
| Grip strength | 21.23 ± 5.67 | 22.09 ± 4.89 | 0.142 |
| SPPB score | 10.94 ± 1.81 | 11.22 ± 1.65 | 0.257 |

Data are mean±SD or number (%). The chi-square or *t*-tests were used to compare the groups, as appropriate. Significant differences between the FT4 groups are highlighted in bold. Abbreviations: TSH, thyroid stimulating hormone; SPPB, short physical performance battery.

**Supplemental Table 3. Clinical characteristics of the study sample, categorized according to serum T3 concentration (N = 134**)

| Parameter | Low T3 | High T3 | *p-*value |
| --- | --- | --- | --- |
|  | (N=108) | (N=26) |  |
| Sarcopenia |  |  | 0.8 |
| Absent | 80 (74.07%) | 18 (69.23%) |  |
| Present | 28 (25.93%) | 8 (30.77%) |  |
| Sex |  |  | 0.355 |
| Male | 18 (16.67%) | 7 (26.92%) |  |
| Female | 90 (83.33%) | 19 (73.08%) |  |
| Age |  |  | 0.939 |
| <70 years | 63 (58.33%) | 16 (61.54%) |  |
| ≥70 years | 45 (41.67%) | 10 (38.46%) |  |
| Free T4 |  |  | 0.653 |
| <1.5 (ng/mL) | 70 (64.81%) | 15 (57.69%) |  |
| ≥1.5 (ng/mL) | 38 (35.19%) | 11 (42.31%) |  |
| TSH |  |  | 0.207 |
| <0.40 (µIU/mL) | 49 (45.37%) | 16 (61.54%) |  |
| ≥0.40 (µIU/mL) | 59 (54.63%) | 10 (38.46%) |  |
|  |  |  |  |
| Age | 68.72 ± 6.66 | 66.69 ± 9.04 | 0.202 |
| Body mass index (kg/m^2^) | 24.43 ± 3.31 | 23.88 ± 2.91 | 0.572 |
| Skeletal muscle mass (kg) | 21.44 ± 4.49 | 21.96 ± 4.72 | 0.624 |
| Skeletal muscle index (kg/m^2^) | 8.68 ± 1.15 | 8.70 ± 1.18 | 0.663 |
| Free T4 (ng/mL) | 1.41 ± 0.24 | 1.45 ± 0.26 | 0.512 |
| TSH (µIU/mL) | 0.91 ± 1.01 | 0.58 ± 0.81 | 0.164 |
| Grip strength | 21.52 ± 5.43 | 21.65 ± 5.36 | 0.962 |
| SPPB score | 11.03 ± 1.77 | 11.12 ± 1.68 | 0.596 |

Data are mean±SD or number (%). The chi-square or *t*-tests were used to compare the groups, as appropriate. Abbreviations: TSH, thyroid stimulating hormone; SPPB, short physical performance battery.
